# *Streptococcus pyogenes* biofilms—formation, biology, and clinical relevance

**DOI:** 10.3389/fcimb.2015.00015

**Published:** 2015-02-11

**Authors:** Tomas Fiedler, Thomas Köller, Bernd Kreikemeyer

**Affiliations:** Institute of Medical Microbiology, Virology, and Hygiene, Rostock University Medical CentreRostock, Germany

**Keywords:** *S. pyogenes*, biofilm, antibiotic resistance, virulence factors, transcriptional regulation

## Abstract

*Streptococcus pyogenes* (group A streptococci, GAS) is an exclusive human bacterial pathogen. The virulence potential of this species is tremendous. Interactions with humans range from asymptomatic carriage over mild and superficial infections of skin and mucosal membranes up to systemic purulent toxic-invasive disease manifestations. Particularly the latter are a severe threat for predisposed patients and lead to significant death tolls worldwide. This places GAS among the most important Gram-positive bacterial pathogens. Many recent reviews have highlighted the GAS repertoire of virulence factors, regulators and regulatory circuits/networks that enable GAS to colonize the host and to deal with all levels of the host immune defense. This covers *in vitro* and *in vivo* studies, including animal infection studies based on mice and more relevant, macaque monkeys. It is now appreciated that GAS, like many other bacterial species, do not necessarily exclusively live in a planktonic lifestyle. GAS is capable of microcolony and biofilm formation on host cells and tissues. We are now beginning to understand that this feature significantly contributes to GAS pathogenesis. In this review we will discuss the current knowledge on GAS biofilm formation, the biofilm-phenotype associated virulence factors, regulatory aspects of biofilm formation, the clinical relevance, and finally contemporary treatment regimens and future treatment options.

## Introduction

*Streptococcus pyogenes* belongs to the serological group A among the streptococci (group A *Streptococcus*, GAS) and is an exclusively human pathogen. GAS causes significant disease worldwide and adds a large burden to national health care systems (Tan et al., 2014). An excellent compilation of data and estimates of the global burden of GAS diseases from 2005 revealed 616 million cases of pharyngitis, 111 million cases of pyoderma and at least 517,000 deaths due to severe invasive diseases and sequelae. This dataset is manifesting the important status of GAS among bacterial pathogens and is an impressive documentation of GAS impact on global mortality and morbidity (Bisno et al., 2005; Carapetis et al., 2005; Ralph and Carapetis, 2013).

Entry ports for GAS after person to person transmission are oral cavity, skin and wounds. In particular, mucosal membranes of the oropharynx and non-intact skin are preferred colonization sites (Cunningham, 2000; Tan et al., 2014). In otherwise healthy individuals, GAS usually causes mild and self-healing purulent infections of mucosal membranes and skin, such as pharyngitis, impetigo and pyoderma. In patients with predispositions such as immune-suppression, diabetes and related diseases, or specific HLA-DR (MHC class II cell surface receptor) subtypes, occasionally severe and invasive life-threatening diseases occur. Necrotizing fasciitis and streptococcal toxic shock syndrome belong to these disease manifestations with high morbidity and mortality rates. Antibiotic therapy is mandatory, even for uncomplicated primary infections, to prevent secondary autoimmune sequelae like rheumatic heart disease or glomerulonephritis (Cunningham, 2000).

GAS is well adapted to its human host, since it is equipped with a large set of virulence factors of all classes. The bacteria express surface proteins and secreted factors leading to (i) immunoglobulin and complement factor degradation (EndoS, Mac, C5a peptidase) and (ii) general complement inhibition (achieved by M protein, capsule expression and Sic), (iii) extracellular matrix and serum protein binding via multiple MSCRAMMS (microbial surface components recognizing adhesive matrix molecules) (M protein, Cpa, Eno, Epf, up to five different firbronectin-binding MSCRAMMS), (iv) dysregulation of coagulation (plasminogen/plasmin binding, streptokinase Ska activity), and (v) cytotoxic and cytolytic activity toward various host cell types (Nga, SLS, SLO). Depending on the presence of phage-related chromosomal islands as variable parts of the accessory genome in the different GAS serotypes, a variable number of superantigens (SpeA-J, SmeZ) is expressed and secreted (Banks et al., 2002; Spaulding et al., 2013).

The presence of individual genes encoding virulence factors is GAS serotype-specific and expression depends on environmental conditions. Transcriptional changes during GAS cultivation and pathogenesis were recently reviewed (Fiedler et al., 2010). A most recent review highlights the importance of GAS virulence factors for disease manifestation and pathogenesis (Walker et al., 2014). As successful pathogen, GAS tightly controls virulence factor gene expression to keep the number of exposed proteins for immune recognition to a minimum. Regulation occurs on multiple levels including the activity of stand-alone transcription regulators and two component signal transduction systems (Kreikemeyer et al., 2003; Patenge et al., 2013), catabolite control (Almengor et al., 2007), control of mRNA decay (Bugrysheva and Scott, 2010), cis- or trans-acting regulation of small non-coding RNAs (Patenge et al., 2013), and quorum sensing (Jimenez and Federle, 2014). How these regulators interact under *in vitro*, *in vivo*-like, and host infection conditions, and how their activities are hierarchically clustered is currently studied intensively (McIver, 2009; Fiedler et al., 2010; Patenge et al., 2013). Information about these regulatory processes allows a better understanding of GAS pathogenic mechanisms and could identify novel levels for interference with anti-infectiva to prevent and/or cure GAS infections.

Apart from the well-studied GAS virulence traits and pathogenesis mechanisms, like host cell adherence/internalization, phagocytosis resistance, escape from phagocytic killing, host cell apoptosis induction and autophagy escape (Walker et al., 2014), the ability of GAS to form micro-colonies and matured biofilms *in vitro* and *in vivo* was just recently appreciated. Biofilms, due to their composition, physiology and physical parameters present a massive danger signal. The host immune defense interacts on all levels to attack these 3 dimensional foreign structures. Some of the above listed genes encoding virulence factors and regulators moved into the focus of GAS biofilm investigation and are discussed in this review.

Furthermore, the specific features of biofilms, i.e., the 3-dimensional structure, the matrix of extracellular polymeric substances, and the lower growth rates and differences in metabolism of the bacteria, cause problems in efficient antibiotic treatment of GAS organized in such structures. Therefore, in this review, we will also discuss potential alternatives to antibiotic treatment of GAS biofilms.

## Clinical relevance of GAS biofilms

GAS was considered a classical extracellular human pathogen for a long time. Numerous studies have evaluated the potential of these bacteria to adhere to and internalize into almost all host cell types, a feature which was discussed as reason for the occurrence of recurrent GAS infections (Facinelli et al., 2001; Podbielski and Kreikemeyer, 2001). However, now it is under debate if recurrence is sufficiently explained by GAS host cell adherence/internalization or if GAS biofilms play a so far underappreciated role. Moreover, the question if GAS biofilms are clinically relevant needs to be addressed. Here we discuss this aspect with a careful look on terminology (microcolony vs. biofilm) and *in vitro* vs. *in vivo* observations and studies.

Particularly the *in vitro* biofilm phenotype was evaluated with isolate collections and for many of the clinically relevant/predominant GAS serotypes under static and flow conditions. In these studies, a significant heterogeneity of this phenotype was noted among strains of a particular serotype (Lembke et al., 2006). Another study revealed 90% of GAS serotypes, from invasive and non-invasive infections, to form biofilms, thereby supporting the notion that this is a trait of individual strains rather than a general serotype attribute (Baldassarri et al., 2006). Moreover, a reduced capacity to internalize into host cells in combination with macrolide-susceptibility was suggested as a strong reason for a biofilm-positive phenotype, as this is a means of protection from antibiotic treatment (Baldassarri et al., 2006). Together these and other facts suggested inclusion of biofilm phenotype data into epidemiological investigations of GAS (Köller et al., 2010).

Generally, two different entry ports could give rise to microcolony formation and the biofilm phenotype. First, GAS can enter new hosts via the oral cavity and establish in the upper respiratory tract. Here, in particular GAS pharyngitis is associated with antibiotic treatment failure leading to multiple infection episodes in affected patients (Facinelli et al., 2001; Podbielski and Kreikemeyer, 2001). Isolates from such cases have a higher tendency toward resistance against macrolide antibiotics in association with the presence of protein F1, a virulence factor supporting host cell internalization (Facinelli et al., 2001). This observation sustains the theory that GAS have an intracellular sanctuary where they persist and hide from eradication by antibiotic treatment and host defense mechanisms. Conley and colleagues rather related antibiotic treatment failure with biofilm formation capacity of GAS (Conley et al., 2003). They showed pharyngitis treatment failure patient isolates to have a biofilm-positive phenotype and increased MBEC (minimum biofilm eradication concentration) for all contemporary antibiotics used to treat acute pharyngitis cases. Moreover, GAS biofilms were found in tonsillar reticulated crypts, isolated from tonsillectomy material (Roberts et al., 2012). Thus, there is a clear link between GAS caused pharyngitis and biofilm formation capacity.

Second, also human skin acts as entry port for these pathogens. Skin from patients with impetigo and atopic dermatitis is a habitat for GAS microcolonies and biofilms (Hirota et al., 1998; Akiyama et al., 2003). Whether GAS microcolonies represent a specific physiological state with own existence or rather a pre-stage of “mature” biofilm is currently unclear. The latter is likely, as microcolonies are surrounded by a FITC-ConA stainable glycocalyx (Akiyama et al., 2003). Cho and Caparon clearly pointed out that GAS forms biofilm-like bacterial communities during soft tissue infection in zebrafish, which largely differ in gene expression patterns from GAS biofilms on abiotic surfaces (Cho and Caparon, 2005).

Further *in vivo* evidence from animal models revealed GAS biofilm formation during otitis media in a chinchilla middle ear infection model. However, biofilm formation was not strictly required for infectivity (Roberts et al., 2010a). In clinical studies in 37% of all non-severe recurrent acute otitis media (RAOM) cases in children GAS was identified as nasopharyngeal biofilm-producing otopathogen (Torretta et al., 2012). The final and most critical development step of biofilm lifestyle is the dispersal stage, which could transform mild and local infections into severe-disseminating diseases (Connolly et al., 2011a).

Apart from the accumulating data on GAS biofilm-mediated antimicrobial resistance toward contemporary antibiotics, leading to treatment failure and recurrent infection episodes, the fact that biofilm grown GAS are naturally competent and thus transformable with foreign DNA (Marks et al., 2014) is of major clinical relevance and concern.

### Conclusion

Although the GAS biofilm phenotype just recently moved into the focus of research, quite a substantial number of studies collected *in vitro*, but more importantly, also *in vivo* evidence that this GAS lifestyle contributes to many diseases caused by GAS. As outlined above, there is in particular compiling data for a role of GAS biofilms during infection of oto-, nasopharyngeal-, and skin-localized human diseases. Thus, the question on the clinical relevance of GAS biofilms is no longer disputable. However, there is a strong requirement for intensification of research in this area and elucidation of better treatment options.

## Virulence factors associated with the GAS biofilm phenotype

To date, more than 50 virulence factors have been described in GAS. Their expression is tightly regulated and fine-tuned in dependence on growth phase and environmental conditions of the bacteria (Fiedler et al., 2010). Consequently, also the biofilm lifestyle of GAS is associated with a specific pattern of virulence factor expression that differs from that of planktonic GAS.

Since biofilm formation in general comprises at least three distinct stages—i.e., (i) initial adherence and microcolony formation, (ii) biofilm maturation with production of a matrix of extracellular polymeric substances (EPS), and (iii) detachment of sessile cells—it is not unexpected that virulence factor expression patterns differ in these respective stages (O'Toole et al., 2000).

### Transcriptome studies on GAS biofilms

The only transcriptome study on GAS biofilms available so far has been carried out with the GAS HSC5 M14 serotype strain. Here, transcript levels in biofilm bacteria were compared to those of planktonic cells in the exponential and stationary growth phase. This analysis revealed an increased abundance of *speB* and *spd*/*mf* transcripts and a lower abundance of *ska* mRNA in biofilm GAS than in bacteria from the exponential phase of planktonic cultures. While M protein expression was more or less constant in biofilm formation, the capsule biosynthesis genes were slightly induced in the maturation phase (Cho and Caparon, 2005). Cho and Caparon propose that in later stages carbohydrate metabolism and capsule biosynthesis are essential to establish a solid biofilm with bacteria encased in a robust matrix of extracellular polysaccharides (Cho and Caparon, 2005). In *S. pyogenes*, the main sugar components in the matrix are L-glucose and D-mannose (Shafreen et al., 2011).

The data of this study are somewhat contradictory to a later work, where expression of several virulence genes in biofilm and planktonic bacteria of the M3 GAS strain MGAS315 has been assessed by qPCR. In contrast to the work of Cho and Caparon, biofilms and planktonic bacteria were exposed to keratinocytes. When grown as biofilms on live keratinocytes for 48 h, downregulation of genes for streptolysins (*sagA*, *slo*), hyaluronic acid capsule biosynthesis (*hasA*), M-protein (*emm3*) and the cysteine protease SpeB (*speB*), was observed while competence-associated *com* genes were upregulated in comparison to planktonic cells exposed to epithelial cells (Marks et al., 2014).

With these limited data sets of only two GAS serotypes and the differential setup of the biofilm assays in the studies of Cho and Caparon (2005) and Marks et al. (2014) it is not possible to deduce any biofilm specific transcriptome yet. Similar studies including more serotypes are needed. Having in mind that the ability to grow in biofilms is rather a strain specific trait and not a GAS serotype attribute (Baldassarri et al., 2006; Köller et al., 2010) it is questionable if a general biofilm transcriptome can be elucidated.

### MSCRAMMS

It has early been recognized that GAS biofilm formation is largely varying between different strains. Certain GAS strains are able to bind to abiotic polystyrene surfaces, while other strains need matrix or serum protein coated surfaces to establish biofilms or are unable to produce biofilms at all (Conley et al., 2003; Lembke et al., 2006). Obviously, adhesive surface structures are needed in GAS biofilms to mediate autoaggregation and attachment of the bacteria to the biotic or abiotic surface (Manetti et al., 2007; Courtney et al., 2009; Oliver-Kozup et al., 2011, 2013). E.g., various GAS strains, representing 8 *emm* types, likely associated with at least 6 different FCT (fibronectin-binding, collagen-binding, T-antigen)-types, have been shown to lose their ability to form biofilms when treated with trypsin, thereby removing trypsin-sensitive surface proteins (Courtney et al., 2009).

Biofilm formation ability of GAS strains seems to be associated with certain M- and FCT-types. This indicates that adhesion and co-aggregation processes mediated by M- or M-like proteins and/or FCT region-encoded pili are essential for the successful establishment of biofilms (Lembke et al., 2006; Manetti et al., 2007, 2010; Edwards et al., 2008; Köller et al., 2010).

**Figure 1:**
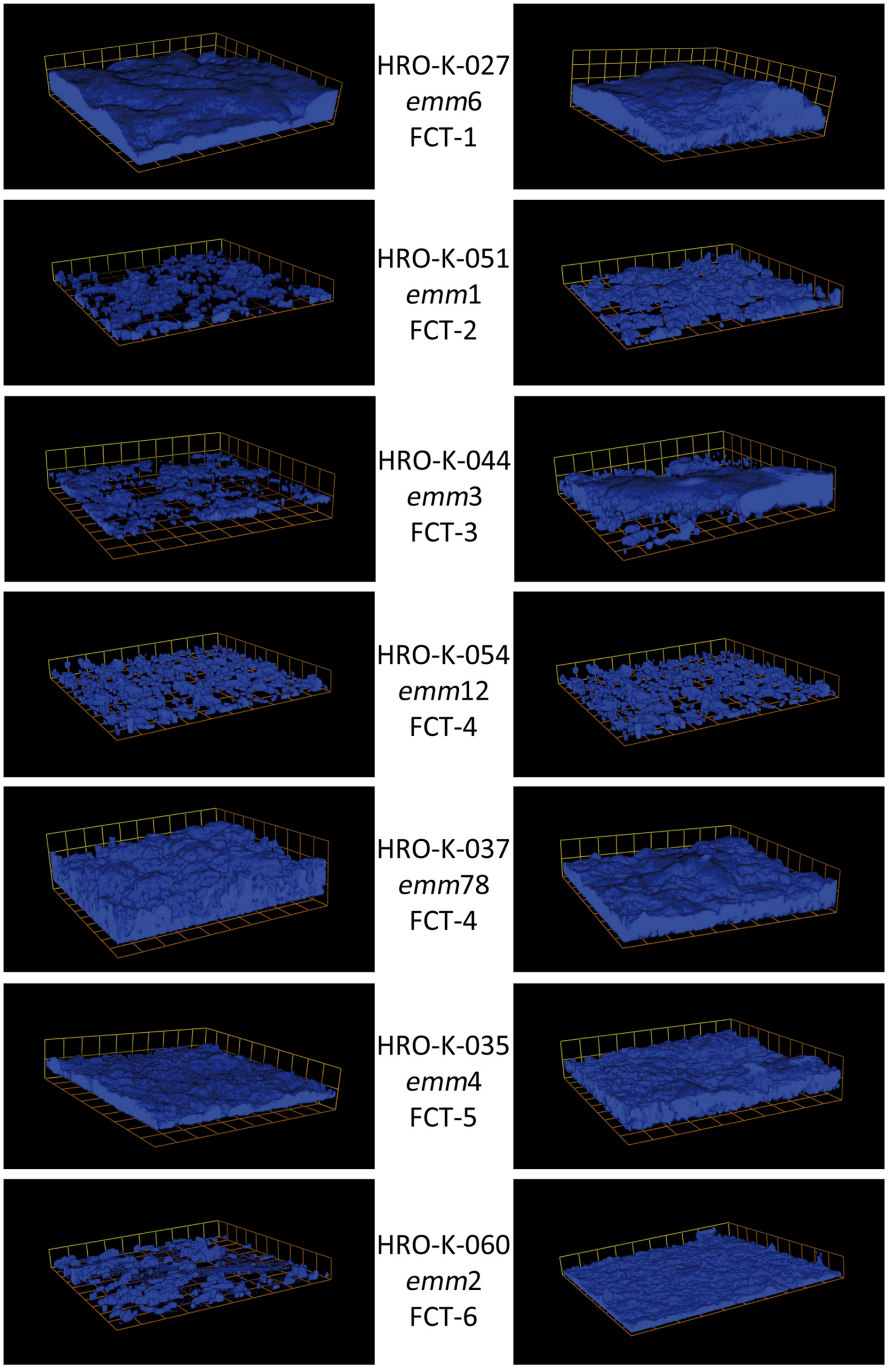
**Three-dimensional images of biofilms of various GAS *emm*/FCT types grown in C-medium in the absence or presence of glucose**. Cells were stained with Alexa Fluor 647 and visualized via Confocal Laser Scanning Microscopy (CLSM); magnification 600 times; box size 13.8 × 13.8 μm. **Left panel**: non-supplemented C-medium. **Right panel**: C-Medium supplemented with 30 mM glucose. Strain names, *emm*-types, and FCT types are given in the middle column.

To date, 9 different types of FCT regions with distinct architectures are described in GAS (Kratovac et al., 2007; Falugi et al., 2008). The FCT region encoded core pilus operon comprises genes for a pilus backbone protein, at least one matrix protein binding ancillary protein, sortases (SrtB/SrtC2), and a signal peptidase (Kratovac et al., 2007; Kreikemeyer et al., 2011). There is clear evidence hinting at an association between FCT type and biofilm formation (Köller et al., 2010; Manetti et al., 2010). While FCT type 1 strains were shown to be generally good biofilm formers, independent of media or pH-conditions, FCT-9 strains are poor biofilm formers under all conditions tested so far. In strains with other FCT types, e.g., FCT-2, FCT-3, FCT-5, and FCT-6, biofilm production depends on culture conditions and is triggered by low pH, e.g., caused by sugar metabolism in unbuffered media (Figure 1). FCT type 4 strains show an inhomogeneous response to environmental conditions with respect to biofilm formation. Generally, M28 and M89 strains tested were poor biofilm formers under all conditions, while M12 strains showed a medium and pH dependent biofilm formation (Köller et al., 2010; Manetti et al., 2010). The specific differences in involvement of pilus structures in biofilm formation in different FCT-type strains might in part be attributed to the high diversity in amino acid sequence of the pilus backbone proteins, which can vary among as well as within FCT-types (Falugi et al., 2008). E.g., T6 type pilus backbone proteins, which can be found in M6/FCT-type 1 strains, seem to strongly promote biofilm formation (Kimura et al., 2012). It has also been demonstrated that ancillary pilus proteins such as Ancillary protein 1 are necessary for biofilm formation (Becherelli et al., 2012).

FCT-region encoded pili are obviously necessary for biofilm formation, but other factors are needed as well, since several GAS strains expressing functional pili in moderate levels are still unable to form biofilms. In contrast, GAS strains with low pilus expression or with defective pili are generally poor biofilm formers (Manetti et al., 2007). As mentioned above, these additional factors are most likely M- and M-like proteins, but also other surface adhesins such as the fibronectin binding proteins PrtF2 and Scl1, or the AgI/II type polypeptide AspA (Cho and Caparon, 2005; Luo et al., 2008; Courtney et al., 2009; Maddocks et al., 2011; Oliver-Kozup et al., 2013). It has been proposed that M- or M-like proteins are needed for LTA-stabilization, thereby increasing hydrophobicity on the GAS surface, which facilitates autoaggregation and adhesion to biotic and abiotic surfaces and consequently biofilm formation (Cho and Caparon, 2005; Courtney et al., 2009). This is supported by the finding that most M- and M-like protein defective mutants show decreased biofilm formation and lower hydrophobicity compared to their wild type parent strains (Cho and Caparon, 2005; Courtney et al., 2009).

The cell wall anchored adhesion AgI/II type polypeptide AspA has been shown to mediate GAS M28 biofilm formation on saliva coated surfaces (Brady et al., 2010; Maddocks et al., 2011, 2012; Hall et al., 2014). AgI/II type proteins bind to salivary glycoproteins. In GAS M28 AspA is proposed to mediate biofilm formation by direct protein-protein interaction with the salivary glycoprotein gp-340 (Maddocks et al., 2011). This is supported by the fact that AspA deficient mutants of GAS M28 show an about 50% reduced biofilm mass when cultivated on gp-340 or saliva-coated surfaces. On uncoated polystyrene surfaces biofilms of AspA mutants resembled those of the cognate WT strains (Maddocks et al., 2011).

The collagen-like protein Scl1 binds cellular fibronectin and also mediates biofilm formation (Caswell et al., 2010; Oliver-Kozup et al., 2011, 2013). M3 strains intrinsically harboring a *scl1* gene with a mutation that results in production of a truncated Scl1 protein where shown to be unable to form biofilms on abiotic surfaces. M41, M28, and M1 strains with *scl1* deletions showed a decreased biofilm formation compared to their cognate wild types. *S. pyogenes* Scl1 expressed on the surface of the heterologous host *Lactococcus lactis* enables biofilm formation of this bacterium (Oliver-Kozup et al., 2011). Considering its specific binding to cellular fibronectin, Scl1 might be of special importance for microcolony/biofilm formation of GAS in wounds (Oliver-Kozup et al., 2013).

Apparently, a critical amount (or number?) of surface associated adhesive structures is necessary to mediate initial adherence and autoaggregation of the bacteria. As mentioned above, structures potentially involved are pili, M- or M-like proteins, PrtF2, Scl1, or AspA. The importance of the respective structures in different GAS strains/M-serotypes/FCT-types obviously differs. Also regulatory mechanisms are not ubiquitous among GAS strains. While in some strains environmental signals such as a low pH are needed to induce biofilm formation, other strains produce biofilms in a pH-independent manner. It is likely that some of the adhesive structures are more important to mediate the initial adherence to abiotic surfaces while others will be crucial for adherence to biotic structures. For sure the latter will be of more significance in the patient. A concerted investigation of all surface structures mentioned above in terms of the involvement in biofilm formation on biotic surfaces is still missing.

### SpeB and other secreted enzymes

The streptococcal pyogenic exotoxin B (SpeB) and other secreted enzymes are associated with the biofilm lifestyle of GAS. SpeB as a secreted cysteine protease is suspected to degrade peptides that stabilize the biofilm matrix (Roberts et al., 2010a,b; Connolly et al., 2011a,b). It has been shown that in the M1 serotype strain MGAS5005 SpeB activity is negatively correlated with biofilm production. While regulator mutants with high SpeB production (MGAS5005Δsrv) are completely unable to form biofilms, deletion of *speB* in these strains reconstitutes the biofilm phenotype of the wild type strain. Also chemical inhibition of SpeB activity increases biofilm masses of MGAS5005Δsrv (Roberts et al., 2010a,b). Furthermore, the external addition of active SpeB to MGAS5005 cultures significantly inhibits biofilm formation (Roberts et al., 2010b).

In line with these data, downregulation of *speB* expression has been observed in biofilms of an M3 serotype strain compared to planktonic bacteria (Marks et al., 2014).

Next to SpeB, other secreted enzymes might be critical for biofilm formation. Proteases and nucleases that potentially degrade components of the extracellular matrix need to be suppressed to maintain the structural integrity of GAS biofilms. While in other streptococci such as *S. intermedius*, *S. mutans*, or *S. pneumoniae*, extracellular DNA (eDNA) is a structural component of biofilm matrix, there is no direct prove for eDNA in GAS biofilms yet (Whitchurch et al., 2002; Montanaro et al., 2011; Domenech et al., 2012). However, increased secretion of enzyme degrading matrix components at a certain point in time might promote dispersion of GAS biofilms and thereby facilitate distribution of GAS within the host. This hypothesis is supported by the data of Cho and Caparon, who found high *speB* expression levels in mature biofilms compared to planktonic bacteria of the HCS5 M14 GAS strain (Cho and Caparon, 2005).

### Capsule

The role of the hyaluronic acid capsule in GAS biofilms is not entirely clear yet. There are somewhat contradictory observations described by different groups. Cho and Caparon observed a slight induction of capsule biosynthesis gene (*has*-operon) transcripts in GAS strain HSC5 during biofilm maturation. Furthermore, they found a mutant defective of capsule biosynthesis to be unable to form a solid biofilm under flow conditions, while under static conditions the biofilm masses were unaffected by this mutation (Cho and Caparon, 2005). Marks and colleagues on the other hand described a decrease in *hasA* transcription in biofilms of MGAS315 (M3) on keratinocytes in comparison to keratinocyte-exposed planktonic bacteria (Marks et al., 2014). Furthermore, there is indirect evidence that capsule production inhibits biofilms, since it has been shown for several GAS strains that *covS* deletion leads to an increased capsule production but also to lower biofilm biomasses (Sugareva et al., 2010). Initially, a thick capsule might mask adhesive surface structures, thereby preventing adhesion and co-aggregation of the bacteria, which would probably rather inhibit biofilm formation. In later stages of biofilm maturation, capsule production could be involved in establishing a robust biofilm matrix, as suggested by Cho and Caparon (2005).

### Conclusion

From all studies introduced above the M protein as one major virulence determinant of GAS seems to support biofilm formation, but secreted proteins and capsule could impair biofilm formation. However, it has to be kept in mind that the data discussed here are all based on *in vitro* experiments that most likely only poorly resemble the *in vivo* situation. Most of the experimental data are based on biofilms formed on abiotic plastic surfaces, sometimes coated with matrix or serum proteins. Only few studies analyzed biofilms grown on epithelial cells (Fiedler et al., 2013; Marks et al., 2014). To our knowledge, device-associated GAS biofilms have never been described in patients. Furthermore, although GAS microcolony formation in the oropharynx has been observed (Diaz et al., 2011; Roberts et al., 2012; Torretta et al., 2012; Woo et al., 2012), it is yet not known whether complex biofilms—as they can be obtained *in vitro*—are actually occurring in patients. Therefore, taken together, the relevance of these *in vitro* data for real infections in patients remains unclear.

## Regulatory aspects of GAS biofilms

The biofilm lifestyle is associated with broad transcriptional changes, affecting the expression levels of about 25% of the GAS genes (Cho and Caparon, 2005). Several transcriptional regulators were shown to be involved in and crucial for the establishment and maintenance of biofilms. From the data available to date, three major regulatory processes can be deduced that facilitate the biofilm lifestyle of GAS:

- Peptide pheromone based quorum sensing mediated by the short hydrophobic peptides SHP2/SHP3 (Chang et al., 2011).
- Repression of secreted and surface associated enzymes such as the cysteine protease SpeB and other proteases and nucleases (Dmitriev et al., 2008; Roberts et al., 2010a; Connolly et al., 2011a; McDowell et al., 2012).
- Induction of surface associated autoaggregative and adhesive structures such as M- and M-like proteins and the FCT region encoded pilus (Cho and Caparon, 2005; Luo et al., 2008; Manetti et al., 2010).

**Figure 2:**
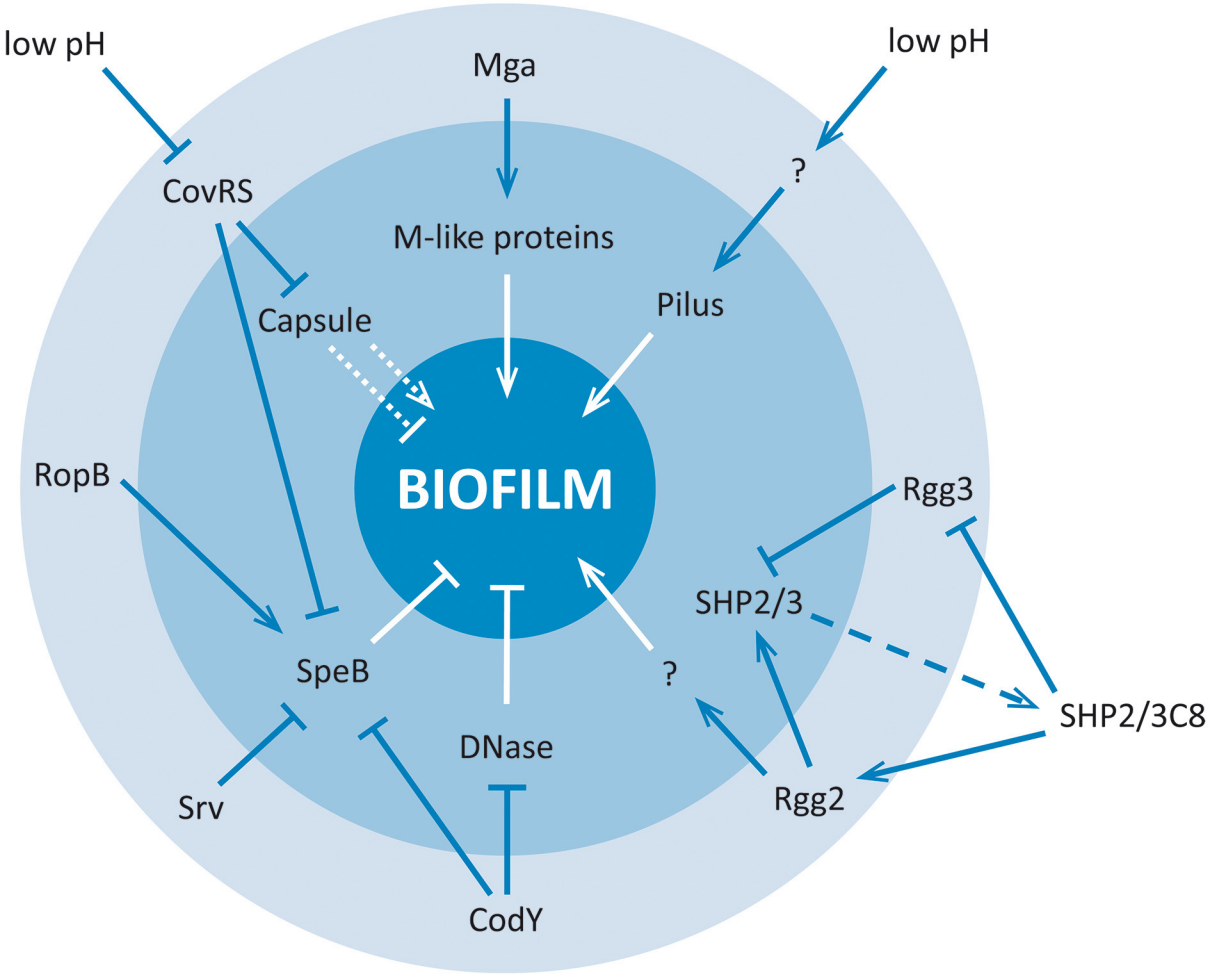
**Regulatory network involved in GAS biofilm formation**. Arrow heads indicate direct or indirect induction, blocked lines indicate direct or indirect repression, dashed lines indicate export out of the bacterial cell, and dotted lines indicate ambiguous effects. Outer circle (light blue): transcriptional regulation level; Inner circle (darker blue): biofilm-associated virulence factors; Outside: environmental conditions and quorum sensing peptides influencing the biofilm phenotype. “?” stands for unknown Regulator/regulatory mechanism.

The major players and the regulatory network contributing to GAS biofilm formation are summarized in Figure 2.

### Quorum sensing

Quorum sensing mechanisms are crucial for biofilm formation in many organisms. In GAS, four different ways of inter- and intraspecies communication are described, i.e., Rgg-, Sil-, lantibiotics-, and LuxS/Autoinduer-2-dependent processes (Jimenez and Federle, 2014).

In GAS, biofilm formation is associated with peptide-pheromone based quorum sensing mediated by the short hydrophobic peptide (SHP) pheromones SHP2 and SHP3. These peptide pheromones are encoded downstream of two genes encoding for the Rgg-like transcriptional regulators Rgg2 and Rgg3, respectively (Chang et al., 2011; Federle, 2012; Lasarre et al., 2013; Aggarwal et al., 2014). The propeptides are secreted and processed to the mature peptide pheromones SHP2C8 and SHP3C8, which are taken up into GAS via the oligopeptide permease Opp. The transcription of both peptide pheromone genes *shp2* and *shp3* is inhibited as long as Rgg3 is bound to the respective promoters. SHP2C8 and SHP3C8 bind to Rgg3 and Rgg2, leading to a dissociation of Rgg3 from and binding of Rgg2 to the *shp2* and *shp3* promoters. In a positive feedback loop, this induces the expression of *shp2* and *shp3* (Chang et al., 2011; Aggarwal et al., 2014). In GAS M49 NZ131 it has been shown that SHP2/3 dependent activation via Rgg2 induces biofilm production, while Rgg3 represses biofilms via repression of SHP2/3 production. It is not known to date, which transcriptional changes are caused by the SHP2/3 dependent activation of Rgg2 and inactivation of Rgg3 that finally lead to biofilm formation. Furthermore, it has not been elucidated yet whether this system also controls biofilm formation in other GAS strains, but *in silico* analyses show that Rgg2 and Rgg3 are present in all GAS strains (Chang et al., 2011).

Two of the other above-mentioned quorum sensing systems of GAS have been associated with the GAS biofilm lifestyle as well. For an M18 strain it could be shown that a SilC deletion mutant was significantly impaired in biofilm formation (Lembke et al., 2006). Furthermore, there are hints that LuxS is involved in the control of SpeB production and *emm* gene expression, which could influence biofilm formation (Lyon et al., 2001; Marouni and Sela, 2003; Siller et al., 2008; Beema Shafreen et al., 2014). Both of the latter QS systems have not been investigated in the context of GAS biofilm in detail yet. For more details on GAS quorum sensing please refer to a current review by Jimenez and Federle (Jimenez and Federle, 2014).

### Transcriptional regulators of SpeB and other secreted enzymes

Since SpeB activity leads to dispersal of biofilm structures and prevents biofilm formation in GAS, repression of *speB* transcription is necessary for successful biofilm establishment (Doern et al., 2009). Therefore, regulators involved in transcription of *speB* also control biofilm formation in GAS. Transcriptional regulation of SpeB is quite complex and involves direct and indirect actions of numerous GAS regulators, as recently reviewed by Carroll and Musser (2011). Positive regulators directly acting at the promoter of the *speB* gene are RopB, another member of the Rgg-regulator family also referred to as Rgg1 (Chaussee et al., 1999; Neely et al., 2003; Dmitriev et al., 2008; Hollands et al., 2008), and the sugar metabolism regulator CcpA (Kietzman and Caparon, 2010; Shelburne et al., 2010). Consequently, deletion of the *ropB* gene leads to lower *speB* expression and an increased biofilm formation as shown in the M49 NZ131 strain (Chang et al., 2011). To our knowledge, for CcpA an influence on biofilm formation has not been elucidated yet.

The CovR (aka CsrR) response regulator of the CovRS two component system probably binds directly to the *speB* promoter as well, acting as a transcriptional repressor (Miller et al., 2001). Consequently, repression of *speB* transcription by CovR enables GAS biofilm formation. CovRS influence on biofilm formation seems to be serotype or even strain dependent. It has been shown that deletion of the sensor kinase CovS leads to decreased biofilm formation in most strains tested. However, for some M6 strains an increased biofilm formation has been observed in CovS deletion strains (Hollands et al., 2010; Sugareva et al., 2010). Furthermore, it was shown that a mutant of the HSC5 strain lacking the CovR response regulator is unable to form biofilm at all (Cho and Caparon, 2005).

Another virulence-associated regulator, Srv, is involved in control of of *speB* expression via indirect mechanisms (Reid et al., 2004; Doern et al., 2009; Roberts et al., 2010a; Connolly et al., 2011a). The deletion of *srv* in the M1T1 strain MGAS5005 leads to an increased activity of SpeB and therefore to loss of the biofilm phenotype (Reid et al., 2006; Doern et al., 2009). In Western Blot analyses SpeB could not be detected in MGAS5005 biofilms after 24 h growth, whereas in the *srv* deletion mutant high amounts of SpeB are present in cultures after 24 h growth (Doern et al., 2009). The Srv mediated repression of SpeB activity is not restricted to the MGAS5005 strain, which has a naturally occuring mutation that leads to an inactive CovS sensor kinase. The effects of Srv on SpeB and biofilm production have also been observed for other GAS strains, although effects of *srv* deletion are not as drastic in those strains as they are in MGAS5005 (Connolly et al., 2011a).

Another regulator potentially involved in biofilm formation is CodY, a regulator involved in the response to nutrient deprivation in many gram positive bacteria (Sonenshein, 2005). CodY deletion mutants were shown to have a reduced biofilm formation capacity of GAS in chemically defined medium (McDowell et al., 2012). This effect probably also results from the indirect CodY-mediated repression of the production of SpeB and other secreted proteases and nucleases (McDowell et al., 2012).

### Transcriptional regulation of biofilm-relevant MSCRAMMS

The transcriptional regulation of GAS surface associated adhesins has been subject to extensive investigations and the regulatory networks have often been reviewed in the past (Kreikemeyer et al., 2003; Hondorp and McIver, 2007; McIver, 2009; Fiedler et al., 2010). Nevertheless, only few of the regulators involved have been investigated with respect to their impact on biofilm formation. Since biofilm formation is apparently associated with the pilus and the M-protein family, it is quite obvious that transcriptional regulators influencing the expression of the FCT region encoded pilus genes and the *emm* gene should influence biofilm formation in GAS. Mga is the major stand-alone transcriptional positive regulator of *emm* and *emm*-like genes (Hondorp and McIver, 2007). Consequently, Mga inactivation leads to a loss of autoaggregation and biofilm formation capacity in GAS (Cho and Caparon, 2005; Luo et al., 2008). Regulation of Mga itself is very complex and was recently reviewed (Hondorp and McIver, 2007; Patenge et al., 2013).

**Figure 3:**
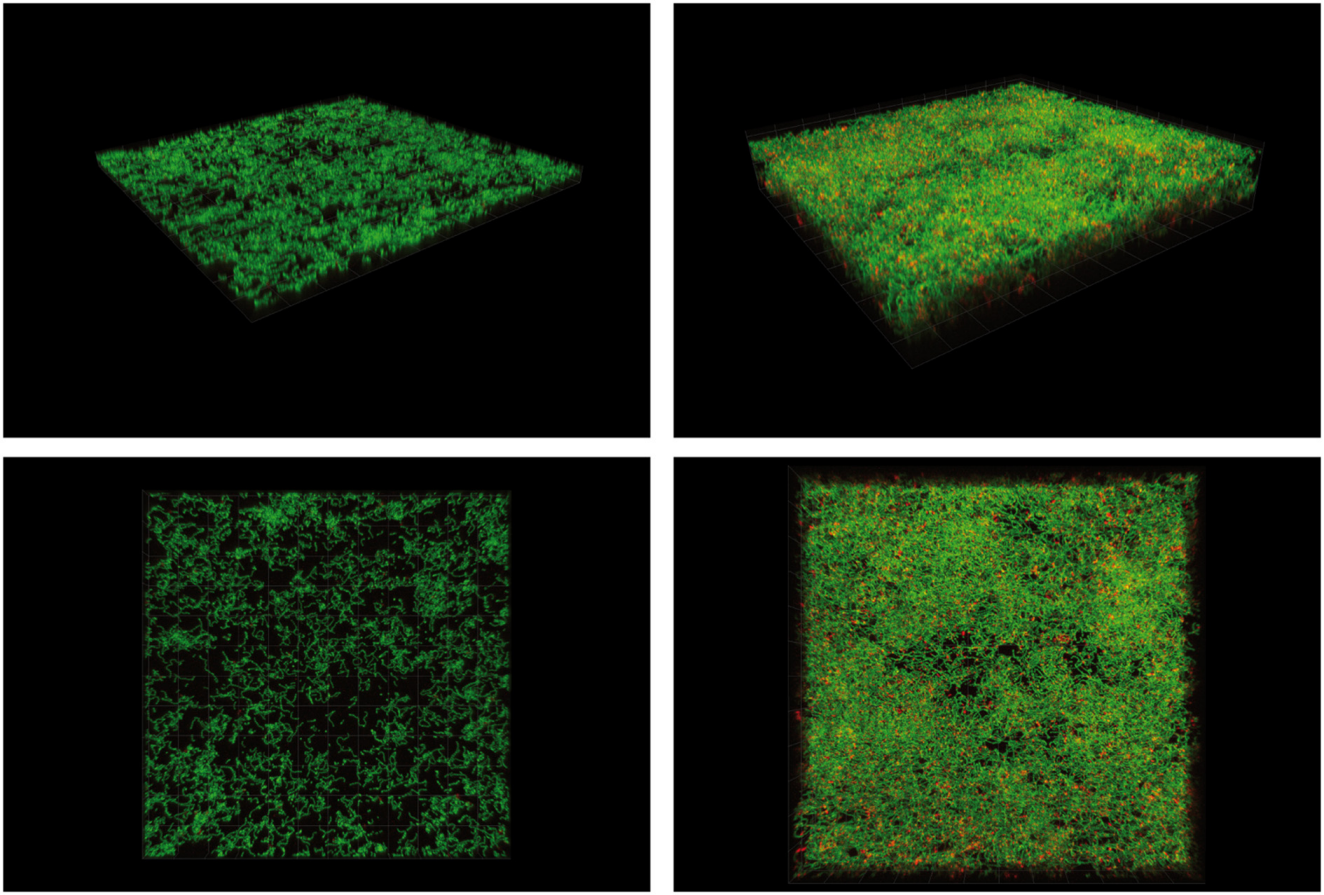
**Confocal Laser Scanning micrographs of 24 h *emm3*/FCT-3 GAS strain HRO-K-044 biofilms cultured in alkalined or acidified C-medium**. Cells were stained with live/dead dye containing Syto9 and Propidiumiodide. Magnification 630 times; box size: 19.8 × 19.8 μm. **Left panel:** mature biofilm grown in C-medium with initial pH of 8.5. **Right panel:** mature biofilm grown in C-medium with initial pH of 6.5. Upper row: 45° perspective. Lower row: top view.

For some strains, i.e., those harboring an FCT-2, -3, or -4 type pilus encoding region, one of the major environmental signals driving biofilm formation is the external pH, as shown exemplarily for an FCT type 3 strain in Figure 3. In these strains, pilus expression is induced under acidic conditions. In contrast, FCT-1 strains produce pH-independent biofilms and do not show any pH-dependent differences in pilus gene expression (Köller et al., 2010; Manetti et al., 2010). The regulator(s) mediating the pH-driven expression of the pilus genes are not known yet. It is likely that the FCT-region encoded RofA-like regulators RofA or Nra might be involved, although this has not been experimentally proven yet (Kreikemeyer et al., 2002, 2011).

#### Conclusion

Environmental signals such as low pH and critical levels of peptide pheromones initiate complex regulatory circuits leading to biofilm formation in GAS. The details in environmental triggers, transcriptional changes, and regulators involved seem to be strain-specific and are not completely understood yet.

## Treatment of GAS biofilms—clinical and experimental aspects

Penicillin remains the most important therapeutic agent to medicate GAS skin and upper respiratory tract infections such as impetigo, erysipelas, cellulitis, tonsillitis, pharyngitis, or scarlet fever (Bisno et al., 2002; Shulman et al., 2012). Fortunately, there is no confirmed case of beta-lactam resistant GAS to date. Patients with penicillin intolerance can be treated with cephalosporins or macrolides such as erythromycin, azithromycin or clarithromycin instead. Of note, increasing numbers of infections with macrolide-resistant strains are reported, ranging up to 38% of erythromycin resistant isolates (Syrogiannopoulos et al., 2001; Sauermann et al., 2003; Richter et al., 2005; Ardanuy et al., 2010; Rubio-Lopez et al., 2012). This leads to a decrease in suitability of this agent class for calculated treatment of local GAS infection.

Severe systemic GAS infections such as sepsis, necrotizing fasciitis or streptococcal toxic shock like syndrome (STSS) are most commonly treated utilizing penecillin in combination with clindamycin. The combination with clindamycin proved to be beneficial to prevent GAS toxin production during therapy (Zimbelman et al., 1999; Russell and Pachorek, 2000).

Although there seems to be no naturally occurring penicillin resistant GAS isolates, it was stated that in about 30% of GAS infections the pathogens are not completely eradicated despite adequate antibiotic therapy and general susceptibility of the GAS isolates (minimal inhibitory concentration [MIC] ≤ 0.25 mg/L), potentially resulting in recurrent infections and persistent carriage (Conley et al., 2003; Baldassarri et al., 2006; Ogawa et al., 2011). There are two explanations discussed in the literature. On the one hand, intracellular persistence of GAS could prevent successful eradication of the bacteria by antibiotic treatment of the patients (Podbielski and Kreikemeyer, 2001). On the other hand, biofilms are discussed to be a cause for treatment failure. It was shown that GAS isolates organized in biofilm-structures could suffer penicillin concentrations up to 400 mg/l (minimum biofilm eradication concentration [MBEC] ≥ 400 mg/L). This exceeds the usual MICs by far, displaying clinical resistant levels (Conley et al., 2003; Baldassarri et al., 2006; Lembke et al., 2006; Shen et al., 2013). Hence, GAS organized in biofilm structures are able to survive antibiotic treatment that is adequate for planktonic GAS. To reach a better clinical outcome and lower treatment failure rates, it would be necessary to adapt treatment of GAS infections to achieve an effective degradation of biofilm structure.

### Experimental approaches of GAS biofilm treatment

Degradation of bacterial biofilm structures can be achieved by treatment with endolysins. This has been reported for several gram positive pathogens, e.g., *Staphylococcus aureus*, *Streptococcus pneumoniae*, *Bacillus antracis*, *Streptoccus suis* (Loeffler et al., 2001; Schuch et al., 2002; Becker et al., 2008; Domenech et al., 2011). Even species with a high tendency to multiple antibiotic resistances can be efficiently killed by such lysins.

For degradation of GAS biofilms, the streptococcal-specific bacteriophage C1 encoded bacteriophage lysin C (PlyC) is of special interest. It was shown that this multimeric N-acetylmuramoyl-L-alanine amidase hydrolyzes GAS cell walls and eliminates bacterial cells *in vitro* and *in vivo* (Krause, 1957; Fischetti et al., 1971; Raina, 1981; Loeffler et al., 2001; Nelson et al., 2001, 2006; Köller et al., 2008). Shen and colleagues furthermore demonstrated PlyC to degrade both GAS biofilm structures and biofilm associated cells efficiently, thereby affecting GAS biofilms significantly more than penicillin (Shen et al., 2013). These abilities make PlyC a reasonable candidate supplement for therapeutic treatment of GAS infections. However, more *in vitro*, *in vivo*, and clinical studies are needed to elucidate the applicability of PlyC for GAS biofilm eradication in patients.

Next to this endolysin, other substances with known broad antibacterial properties have been shown to inhibit GAS biofilms. Of special interest could be manuka honey that is available as sterilized medical grade honey (medihoney) for topic wound treatment. Maddocks and others described medihoney to inhibit the expression of *sof* and *sfb1* genes encoding for fibronectin binding streptococcal surface proteins, resulting in reduced human tissue binding and biofilm formation capacity (Maddocks et al., 2012). It has to be considered that these findings have been reported only for one clinical isolate representing an M28 serotype (MGAS6180; M28). It remains to be seen if these effects are transferable to other strains/*emm* genotypes, since there are remarkable differences of the regulatory networks reported as discussed above.

It has furthermore been shown that the fatty acid messenger cis-2-decenoic acid produced by *Pseudomonas aeruginosa* can induce dispersion of biofilms of GAS and other bacteria. If such substances could be administered in patients in combination with conventional antibiotics, this would probably lead to eradication of GAS biofilm associated infections (Davies and Marques, 2009).

Beside the chemical treatment of mature GAS biofilms, also probiotic effects of physiological bacteria on GAS biofilm formation have been reported (Guglielmetti et al., 2010a,b; Fiedler et al., 2013). It has been shown that bacteria physiologically colonizing the upper respiratory tract, such as *Streptococcus oralis* and *Streptococcus salivarius*, protect epithelial cells from GAS adherence. This observation indicates a role of these bacteria in host health. It was reported that *S. oralis* could induce protection of eukaryotic cells even without largely binding to the cells or producing bacteriocins affecting GAS (Fiedler et al., 2013). Further on, *S. salivarius* was shown to provide host cell protection against GAS by forming an impermeable biofilm so the host epithelial cells are inaccessible for initial GAS tissue colonization (Fiedler et al., 2013). Other authors stated *S. salivarius* K12 to antagonize GAS growth by expressing the lantibiotics salivarin A2 and B subsequently influencing GAS biofilm formation (Di et al., 2013, 2014).

### Conclusion

The standard antibiotic medication for patients with GAS infections is not sufficient to eradicate GAS biofilms. Alternative or additional therapeutics are currently investigated. Phage lysin C represents the most promising candidate for clinical application. However, more efforts are needed in developing treatment strategies to prevent extensive and repeated antibiotic treatment in patients with biofilm associated recurrent GAS infections.

## Outlook and future challenges

Due to the high tolerance of GAS biofilms toward antibiotics, GAS biofilms are likely to be associated with antibiotic treatment failure in patients. Therefore, the major future challenge will be the development of new therapeutic strategies to prevent the extensive use of antibiotics on patients with recurrent GAS biofilm associated infections. As can be seen from this review, we are just at the beginning of understanding the GAS biofilm phenotype and its relevance for GAS pathogenesis. Therefore, extensive further studies on the biological processes involved in GAS biofilm formation are necessary.

A major question in this context is which environmental factors trigger GAS biofilm formation. It is highly likely that apart from carbon source, external pH, and peptide pheromone levels, host innate immune responses trigger GAS biofilm development *in vivo*. Furthermore, more efforts are needed to decipher the role of individual virulence factors and gene regulation circuits in GAS biofilm development *in vivo*, since most of the current knowledge is based on *in vitro* data.

Since the GAS biofilm formation capacity is very strain specific, it will be important to include the determination of the biofilm phenotype of GAS strains into epidemiological investigations. Particularly the relation of the biofilm phenotype to other parameters frequently studied in GAS epidemiology, e.g., *emm*- or FCT-type, antibiotic resistance or presence/absence of certain virulence factors, needs to be elucidated. Ideally, such studies will lead to phenotypic profiles that will allow deducing the potential of GAS isolates for biofilm formation.

Such tools would facilitate the specific treatment of patients with recurrent infection potentially associated with GAS biofilms. The most promising candidates for clinical application in GAS biofilm eradication in patients are specific phage lysins such as PlyC, since they have excellent MBEC values. Research in this area should be intensified toward application in clinical practice.

### Conflict of interest statement

The authors declare that the research was conducted in the absence of any commercial or financial relationships that could be construed as a potential conflict of interest.
